# A painless erythematous swelling of the external ear as a manifestation of Lyme disease: a case report

**DOI:** 10.1186/s13256-020-02377-x

**Published:** 2020-04-16

**Authors:** Allison Remiker, David Haslam, Theodosia A. Kalfa

**Affiliations:** 1 Ann & Robert H. Lurie Children’s Hospital of Chicago, 225 E. Chicago Ave., Box 30, Chicago, IL 60611 USA; 2 Cancer and Blood Diseases Institute, Cincinnati Children’s Hospital Medical Center, 3333 Burnet Avenue, MLC 7015, Cincinnati, OH 45229-3039 USA; 3 Division of Infectious Diseases, Cincinnati Children’s Hospital Medical Center, 3333 Burnet Avenue, Cincinnati, OH 45229-3039 USA

**Keywords:** Borrelial lymphocytoma, Lyme disease, External ear erythema

## Abstract

**Background:**

Lyme disease is the most common tick-borne illness in the USA, Canada, and Europe. Clinical manifestations vary greatly, with localized skin findings functioning as early signs of the disease, followed by disseminated disease. The rarest dermatologic presentation of Lyme is a borrelial lymphocytoma, occurring distinctly in Europe and caused typically by *Borrelia afzelii*.

**Case presentation:**

We report a case of a Caucasian 5-year-old European-American boy with slowly progressing, painless edema and erythema of his right pinna. Travel history revealed significant exposure to ticks during a recent trip to Eastern Europe. Laboratory testing for *Borrelia burgdorferi* demonstrated mixed positivity. He was treated with a 21-day course of amoxicillin, with complete resolution of symptoms and no sign of secondary Lyme disease.

**Conclusions:**

Borrelial lymphocytoma is a rare manifestation of Lyme disease in North America, although not uncommon in Europe. Diagnosis is made by the presence of a painless erythematous swelling typically found on the ear lobe, nipples, or testes. Laboratory tests are available but with low sensitivity, therefore, a high index of suspicion is necessary for a clinical diagnosis to be made. Treatment for isolated borrelial lymphocytoma is doxycycline 4 mg/kg up to 100 mg twice daily, whereas for children less than 8 years of age amoxicillin 50 mg/kg divided three times daily, for 3–4 weeks, is preferred.

## Background

Lyme disease is endemic in North America, and is the most common cause of tick-borne illness in the USA, Canada, and Europe [1]. Zoonotic transmission occurs after tick bites from various *Ixodes* species. Typical species include *Ixodes scapularis* (black-legged tick or deer tick) on the East coast of North America and *Ixodes pacificus* (western black-legged tick) on the West coast in North America, whereas *Ixodes ricinus* (sheep tick) is most common in Europe. Infections are caused by species of the bacterial spirochete family, *Borreliaceae*. In North America, the most predominant culprit of disease is *Borrelia burgdorferi*; in Europe, the most predominant culprits of disease are *Borrelia afzelii* and *Borrelia garinii* [2].

Clinical manifestations of disease typically involve the skin and cardiovascular, musculoskeletal, and nervous systems. Hematogenous dissemination of bacteria occurs within days to weeks following a tick bite; host-driven immune responses often lead to specific symptoms [1]. Borrelial lymphocytoma (BL) as a skin manifestation of Lyme disease overall is rare, found in only 5% of patient cases, and is atypical in North America [3]. We describe a case of a young boy residing in North America with slowly progressing, painless edema and erythema of the right pinna consistent with BL. Early diagnosis and treatment of infection is critical to avoid ramifications of late disseminated disease.

## Case presentation

A 5-year-old Caucasian European-American boy was referred to Hematology due to concern for a possible autoimmune disorder because of slowly progressing, painless edema and erythema of his right pinna. He was seen recently by Otolaryngology and a primary ear disease was ruled out. Since he had history of immune thrombocytopenia (ITP) at 3 years of age, a concern for an underlying autoimmune disorder was raised. He was in his normal state of health until 4 months prior (in September), when he was noted to have patchy red swelling of his right external ear with no associated pruritus or pain. Initially the erythema waxed and waned; it only lasted hours at a time. However, for the preceding 2 months it had become unremitting. Associated signs included an evanescent circular red rash on his left cheek, also for the last 4 months. He did not experience any constitutional or musculoskeletal symptoms.

**Fig. 1:**
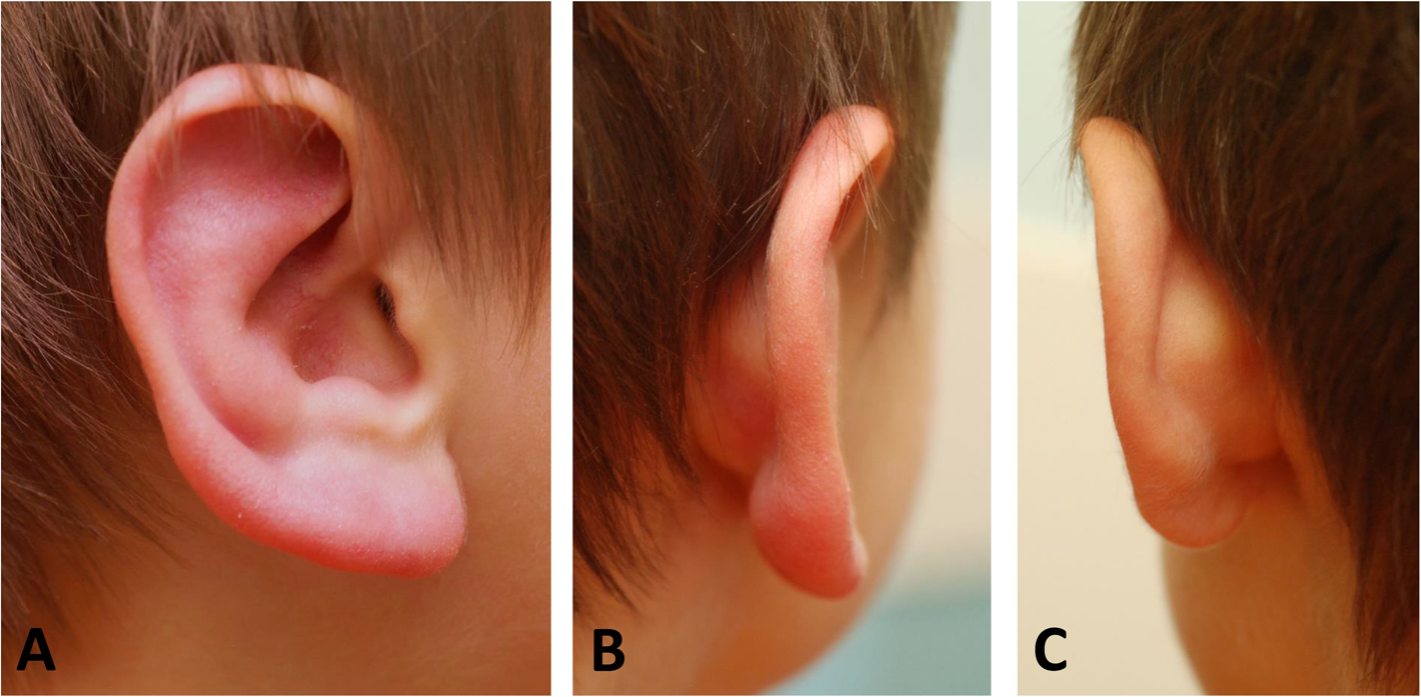
**a/b.** Painless edematous and erythematous right auricle. **c.** Comparison of the unaffected left auricle

On examination, his vital signs were normal for age: temperature 36.9 °C, pulse 124 beats per minute, blood pressure 106/65, weight 11.9 kg, and height 85.2 cm. He appeared well with the examination most notable for an erythematous, edematous right auricle (Fig. 1), which was non-tender to palpation. His left auricle was unaffected and normal in appearance. There were no additional skin findings including rash, bruising, or petechiae. His ear canals were normal with pearl-gray colored tympanic membranes containing no fluid. He was well nourished and in no acute distress. His head was normocephalic. His eyes had normal reactive pupils, no discharge, no erythema or swelling, and no scleral icterus. His nose was normal in appearance. His oropharynx was moist with normal tonsils. His neck was supple with minor bilateral cervical lymphadenopathy. His lungs were clear to auscultation without respiratory distress. A cardiac examination noted a regular rate and rhythm, normal S1 and S2, without murmur. There was no axillary lymphadenopathy. An abdominal examination revealed a soft and non-tender abdomen without organomegaly. There was no inguinal lymphadenopathy. A neurological examination demonstrated an alert child with appropriate responsiveness for age, normal muscle tone, and no weakness. His extremities showed no deformities, joint abnormalities, or edema.

Initially we considered a broad differential diagnosis with this case of painless erythema and swelling of the ear lobule and concha, in the setting of an associated erythematous patch of the cheek. We evaluated for possible mastocytosis with the ear as a location for a hive-like rash. However, the lesion was non-pruritic in nature, and serum tryptase was normal (3.4 mcg/L). We also considered autoimmune and/or rheumatologic conditions. However, a complete blood count with differential (white blood cell 8.9 K/mcL, hemoglobin 11.8 gm/dL, platelet 347 K/mcL, absolute neutrophil count 3.92 K/mcL, absolute lymphocyte count 3.83 K/mcL), lymphocyte subpopulations (CD3 3304 cells/mcL, CD4 1835 cells/mcL, CD8 1300 cells/mcL, CD19 671 cells/mcL, CD16/56,316 cells/mcL, CD4:CD8 1.4), serum immunoglobulins (IgA 144 mg/dL, IgE 72 international units/mL, IgG 1260 mg/dL, IgM 57 mg/dL), and complement C3 109 mg/dL and C4 17.1 mg/dL levels were normal, and anti-nuclear antibodies (ANAs) and an anti-extractable nuclear antigen (ENA) panel (JO-1 antibody, SSB, SSA, anti-Smith, ribonucleoprotein) were negative.

Of interest, his travel history revealed that he had spent 2 months in the Czech Republic over the summer, in a heavily wooded area, and was bit by a significant number of ticks before he returned to the USA just a few weeks prior to the initiation of symptoms.

Collectively, the presence of the auricular lesion, cervical lymphadenopathy, an associated lesion suspicious for erythema migrans (EM), and history of tick exposure led to the possibility of BL. An enzyme-linked immunosorbent assay (ELISA) test for antibodies against *B. burgdorferi* was positive, although Western blotting for IgM antibodies had 0 of 10 bands and for IgG antibodies 3 of 10 bands, with 5 bands indicating a positive result.

Due to sufficient clinical suspicion, he was placed on amoxicillin 50 mg/kg per day for a 21-day course. Within days of starting the antibiotic, both erythema and edema of the affected area were improved and eventually resolved. The Infectious Disease service was consulted, and on 3-month follow-up his symptoms resolved, and he showed no signs of secondary Lyme disease. A skin biopsy with polymerase chain reaction (PCR) was considered; however, due to clinical improvement this was deemed unnecessary given low yield to identify the organism after successful antibiotic treatment. Nine months following initial diagnosis he remained asymptomatic without return of auricular swelling or new skin, muscle, joint, or neurological findings.

## Discussion

We report here the case of a 5-year-old pediatric patient with slowly progressing, painless edema and erythema of the right pinna diagnosed with BL. He had subsequent resolution of auricular inflammation with antibiotic treatment for Lyme disease without sequelae of secondary Lyme disease.

BL is a rare manifestation of Lyme disease in North America [4], but not uncommon in Europe where the *Ixodes ricinus* tick is endemic. To the best of the authors’ knowledge there are no recent case reports in North America, indicating the paucity of patients with this clinical manifestation and lack of clinical recognition. The present case report highlights that the recognition of a rather common infection, such as Lyme disease, may be obscured when the underlying species is not endemic to the area.

Although *B. burgdorferi* is the typical causative species for Lyme disease in North America, *B. afzelii* is the most predominant species associated with BL in Europe, probably explaining the negative Western blot for IgM and IgG against *B. burgdorferi*. There is currently no available laboratory test for *B. afzelii* in the USA and testing of cases with BL in Europe report sensitivity as low as 40% [5]. In general, utilization of indirect pathogen detection by a two-tiered diagnostic approach of immunoassay followed by confirmation assay or immunoblot is recommended for diagnosis of Lyme borreliosis. If the findings are ambiguous, direct pathogen detection by culture or nucleic acid amplification techniques, typically PCR, have been confirmatory in cases of BL. However, clinical suspicion is essential as the recovery rate of organism is thought to be < 25% in biopsy, with PCR positivity in at most two-thirds of patients. In general, if solitary BL is suspected, initiation of antibiotics is recommended to avoid disseminated disease [6, 7]. In the current patient case, given the significant rapid improvement of the lesion, a skin biopsy was not pursued due to concern of low yield of active pathogen presence. Albeit with its limitations, molecular techniques have been crucial in cases of unusual presentation or failure of treatment [4, 8–10].

BL is a subtype of a heterogenous group of benign lymphoproliferative conditions called cutaneous pseudolymphomas. This set of conditions refers to a predominantly lymphocyte-derived inflammatory infiltrate, which may encompass additional inflammatory cells. BL is caused by B cell lymphocytic infiltration of the dermis and subcutis, producing discoloration and edema of the affected site, often at the location of the tick bite. It characteristically presents as a 1–5 cm purple or red-colored plaque or nodule, most commonly at the ear lobe, nipples, or testes as the organism prefers tissues with somewhat cooler temperature [11, 12]. Consistent with this case presentation, along with a case series, BL is most commonly found in the ear lobe of children, whereas a nipple location is more common in adults [3, 5]. On histologic examination, the infiltrate is accompanied by mixed inflammatory cells including histiocytes, eosinophils, and plasma cells, and, importantly, some cases may exhibit features of B cell lymphoma. Immunohistological staining reveals nodular CD20+ B cell clusters with germinal centers and high proliferation rates, CD3+ T cells, and equally expressed κ and λ immunoglobulin light chains in plasma cells [13].

Typically, symptoms appear within days to 6 months following a tick bite, and most frequently within the months of August or September [14]. The presentation of symptoms without close proximity to the time of the tick bite can lead to delay in diagnosis or poor recognition, as in this patient’s case. Associated signs include concomitant solitary EM, which when present along with lymphocytoma is pathognomonic for BL [15]. However, in comparison to EM, BL often develops later with an extensive or chronic duration [12]. This presentation was consistent with our patient, in addition to regional lymphadenopathy which is commonly found. The treatment for isolated BL is doxycycline 4 mg/kg up to 100 mg twice daily, whereas for children under 8 years of age amoxicillin 50 mg/kg divided three times daily, for 3–4 weeks, is preferred. In refractory cases, intralesional interferon-alpha therapy has been tolerated and successful [8].

## Conclusion

BL is a rare dermatological manifestation of Lyme disease. It is rarely, if ever, seen in North America due to its association with borrelial species more commonly found in Europe. Diagnosis is made by the presence of a painless erythematous swelling typically found on the ear lobe, nipples, or testes. Laboratory testing is available, however, it is limited by low sensitivity. Prompt diagnosis and treatment initiation is essential to avoid ramifications of late disseminated Lyme disease. This case highlights the importance of recognition of rare manifestations of a common disease, in particular, in those with significant travel history.

## Data Availability

Not applicable.

## Abbreviations

ITP: Immune thrombocytopenia
ANA: Anti-nuclear antibodies
ENA: Extractable nuclear antigen
BL: Borrelial lymphocytoma
ELISA: Enzyme-linked immunosorbent assay
EM: Erythema migrans
PCR: Polymerase chain reaction
